# Locally Acquired Human Infection with Swine-Origin Influenza A(H3N2) Variant Virus, Australia, 2018

**DOI:** 10.3201/eid2601.191144

**Published:** 2020-01

**Authors:** Yi-Mo Deng, Frank Y.K. Wong, Natalie Spirason, Matthew Kaye, Rebecca Beazley, Migue L.l Grau, Songhua Shan, Vittoria Stevens, Kanta Subbarao, Sheena Sullivan, Ian G. Barr, Vijaykrishna Dhanasekaran

**Affiliations:** World Health Organization Collaborating Centre for Reference and Research on Influenza, Melbourne, Victoria, Australia (Y.-M. Deng, N. Spirason, M. Kaye, K. Subbarao, S. Sullivan, I.G. Barr, V. Dhanasekaran);; CSIRO Australian Animal Health Laboratory, Geelong, Victoria, Australia (F.Y.K. Wong, S. Shan, V. Stevens);; South Australian Department of Health and Wellbeing, Adelaide, South Australia, Australia (R. Beazley);; Monash University, Melbourne (M.L. Grau, V. Dhanasekaran);; University of Melbourne, Melbourne (S. Sullivan, I.G. Barr)

**Keywords:** Influenza virus, H3N2v, influenza A(H1N1)pdm09 virus, pH1N1, swine influenza, zoonoses, influenza surveillance, pandemic influenza, viruses, Australia, influenza, respiratory infections

## Abstract

In 2018, a 15-year-old female adolescent in Australia was infected with swine influenza A(H3N2) variant virus. The virus contained hemagglutinin and neuraminidase genes derived from 1990s-like human seasonal viruses and internal protein genes from influenza A(H1N1)pdm09 virus, highlighting the potential risk that swine influenza A virus poses to human health in Australia.

Long-term circulation of influenza A viruses (IAVs) among swine poses a public health threat. The 2009 pandemic was caused by a reassortant swine influenza A(H1N1) virus with genes that originated from human and avian IAVs that had circulated among swine for several years (*1*,*2*). Since then, globally enhanced influenza surveillance among swine has indicated continuous introduction of human seasonal influenza viruses into swine, followed by reassortment with influenza A viruses endemic in swine (IAV-S) and persistence of many lineages in swine for several decades (*3*). Although IAV-S are normally limited to transmission among swine, since 2010, a total of 430 cases of human infection with swine-origin influenza A(H3N2) variant viruses (H3N2v) have been detected in the United States (*4*), primarily in young persons exposed to swine at agricultural fairs. Most patients had self-limited influenza-like illness (*5*). Recent data also suggest that IAV-S have been endemic to Australia for many decades, including viruses that were originally derived from human H3N2 viruses as early as 1968, pre-2009 seasonal H1N1 viruses, and influenza A(H1N1)pdm09 (pH1N1) viruses (*6*).

## The Study

In September 2018, a case of human infection with a swine-origin influenza virus was detected in Australia through routine human influenza virus surveillance by the World Health Organization (WHO) Collaborating Centre for Reference and Research on Influenza (Melbourne, Victoria, Australia), which is part of the WHO Global Influenza Surveillance and Response System. The sample was from a 15-year-old female adolescent living in a semirural area in South Australia, ≈100 km from Adelaide. The patient sought outpatient care for a mild respiratory illness ≈8 days after illness onset. The attending physician collected a nasal swab sample and sent it for testing to a laboratory in Adelaide, where influenza A was detected by real-time reverse transcription PCR (RT-PCR) but not subtyped. The sample was subsequently forwarded to the WHO Collaborating Centre for further characterization. It was later determined that the patient had not been vaccinated against influenza in 2018 and had had contact with animals at an agricultural show in South Australia the day before illness onset.

We isolated an influenza A virus in SIAT-1 MDCK cells (*7*) and designated it as A/South Australia/85/2018 (Appendix). Testing of this isolate by real-time RT-PCR with an influenza diagnostic kit from the US Centers for Disease Control and Prevention confirmed that the virus was an influenza A(H3N2) virus with a pH1N1-like nucleoprotein (NP) gene. Hemagglutination inhibition (HI) assays of the isolate showed that this virus had >5-fold antigenic divergence to ferret antiserum raised against human H3N2 viruses collected during 1993–2016 and an H3N2v from the United States (A/Minnesota/11/2010) (Appendix Table 1) (*6*). The A/South Australia/85/2018 virus showed good cross-reactivity (within 2-fold of the homologous HI titer) with pig antiserum to a US swine A(H3N2) virus, A/swine/New York/A01104005/2011 (Appendix Table 2). Although both viruses showed high genetic divergence, they contained a common hemagglutinin (HA) Y155H substitution within antigenic site B (Appendix Table 3); its role in antigenicity remains unknown.

Whole-genome sequencing of A/South Australia/85/2018 indicated that the virus was a 2:6 reassortant of 2 human influenza virus lineages introduced to swine in Australia; the H3 HA and N2 NA genes originated independently from seasonal human influenza viruses that circulated during 1995–1999, and the genes for internal proteins originated from pH1N1 viruses from late 2009 through early 2010. Using a fluorescence-based NA enzyme inhibition assay, we found that A/South Australia/85/2018 was sensitive to 1 class of influenza drugs, the NA inhibitors (oseltamivir and zanamivir); however, it would be expected to be resistant to the adamantane class of drugs (amantadine/rimantadine) because it had an S31N substitution in the matrix (M) 2 gene, which is known to confer high-level resistance to adamantane (*8*).

To identify the evolutionary origins and the epidemiologic links of A/South Australia/85/2018, we further generated the whole and partial genomes of an additional 44 available IAV-S collected in piggeries across several states in Australia during 2013–2018; all were pH1N1-like viruses (Appendix Table 4). These viruses were derived from swine nasal, tracheal, or pooled lung tissue samples submitted on an ad hoc basis for diagnostic investigations during 2012–2018 to the Australian Animal Health Laboratories (Geelong, Victoria, Australia) by commercial piggeries in New South Wales, Queensland, Victoria, and Western Australia.

**Figure 1:**
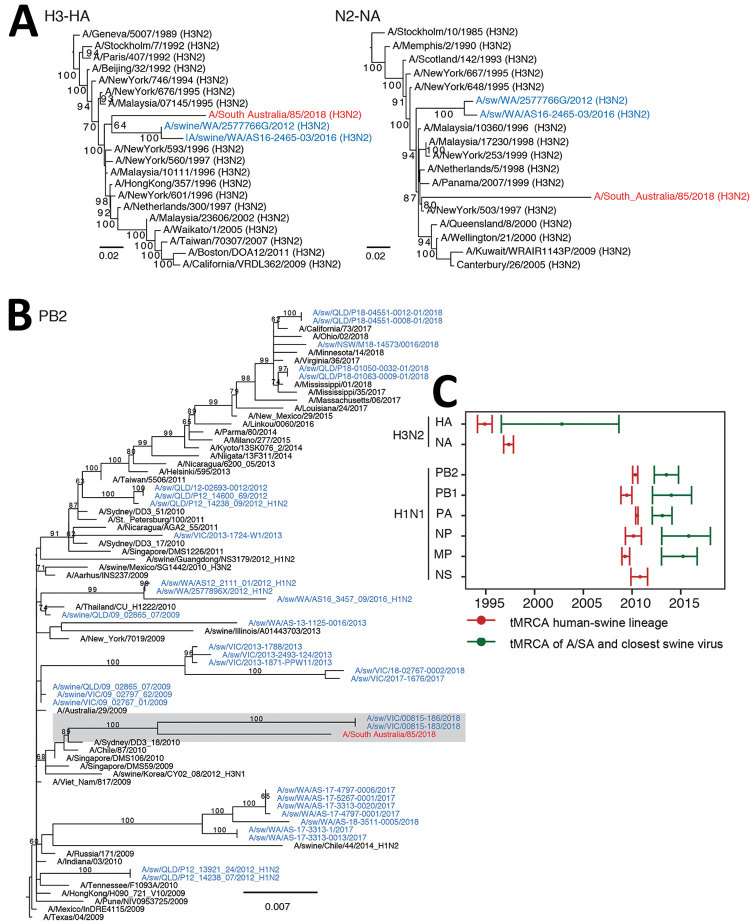
Genetic origin of influenza A/South Australia/85/2018 virus isolated from a human patient in Australia (red) from swine influenza A(H3N2) and H1N1pdm09 viruses. Blue indicates influenza A viruses from swine in Australia. A, B) Maximum-likelihood phylogenies estimated by using RAxML version 8 (*10*) of the HA and NA genes (A) and PB2 gene (B) showing bootstrap values at branch nodes (Appendix). The origins of the remaining 5 internal proteins genes (PB1, PA, NP, MP, and NS) are provided in Appendix Figure 2, and the GenBank accession numbers and dates of sampling are provided in Appendix Table 4. Scale bars indicate nucleotide substitutions per site. C) Calculation of tMRCA. Red indicates means and 95% CIs of the time of origin of each of the Australia swine influenza A virus lineages from human seasonal influenza viruses. Numbers denote viruses that shared the same tMRCA and that formed a similar lineage. Green indicates the time of divergence of A/South Australia/85/2018 from A/swine/WA/2577766G/2012 (H3N2) (for the H3 HA gene) and A/swine/Victoria/18-04095-0003/2018 (H1N1) (for 5 internal protein genes: PB2, PB1, PA, NP, and MP). N2 and NS proteins of A/South Australia/85/2018 are directly derived from human viruses. Divergence times were estimated by using the uncorrelated log-normal relaxed clock model (*11*) in a Bayesian Markov chain Monte Carlo framework in BEAST version 1.10 (https://beast.community). A/SA, A/South Australia/85/2018 virus; HA, hemagglutinin; MP, matrix protein; NA, neuraminidase; NP, nucleoprotein; NS, nonstructural; PA, polymerase acidic; PB, polymerase basic; A(H1N1)pdm09 virus, 2009 pandemic influenza H1N1 virus; tMRCA, time to most recent common ancestor.

Phylogenies inferred with all sequences of swine influenza viruses from Australia, as well as with a representative set of those collected from humans and swine globally (*6*,*9*) (Appendix), showed that the HA gene of A/South Australia/85/2018 was most closely related to swine H3N2 viruses collected from a commercial piggery in Western Australia during 2012–2016 (*6*) (Figure 1, panel A). This H3 swine lineage from Australia was poorly supported (64% bootstrap) and originated from the phylogenetic backbone of human seasonal H3N2 virus, clustering with viruses collected during 1995–1996, whereas their NA was most closely related to that of human H3N2 viruses circulating during 1997–1999 (Appendix Figure 1) and not most closely related to that of swine samples from Australia.

Of note, 5 of 6 internal segments of A/South Australia/85/2018 were most closely related to a swine pH1N1 virus collected in Victoria in 2018 (A/swine/Victoria/00815–183/2018) (Figure 1, panel B). Although there were no IAV-S data from South Australia to trace the immediate origins of A/South Australia/85/2018, these phylogenetic relationships confirm that A/South Australia/85/2018 was acquired locally from swine herds endemically infected with influenza A viruses that had circulated since the mid-1990s and the 2009 H1N1 pandemic.

**Figure 2:**
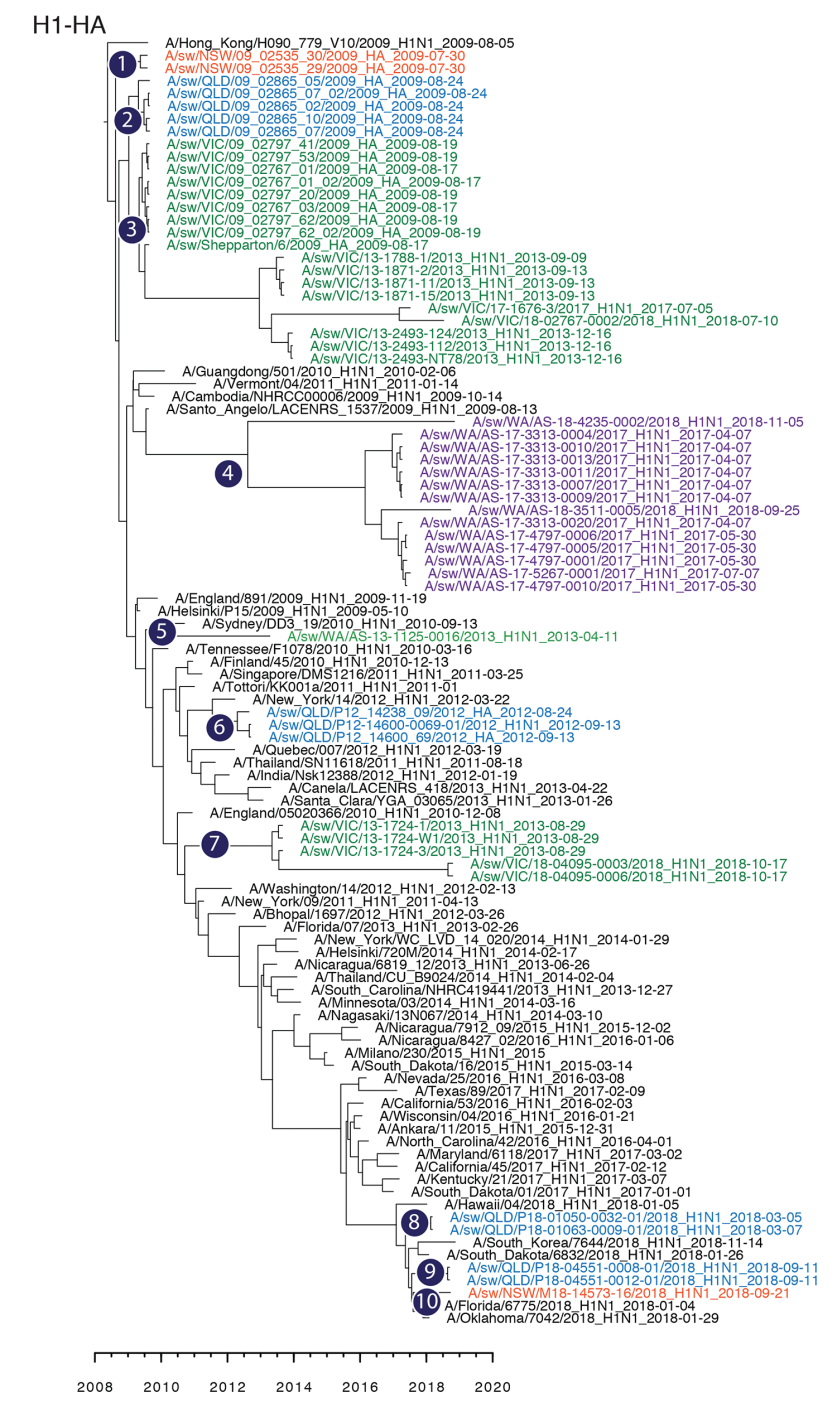
Maximum-clade credibility tree showing the time of emergence from humans and divergence of swine influenza A and influenza A(H1N1)pdm09 virus among swine populations in Australia. Numbers 1–10 denote inferences of individual introductions from humans to swine, and virus names are colored by state of collection: red, New South Wales; blue, Queensland; green, Victoria; purple, Western Australia. Time-scaled phylogenies were estimated by using the uncorrelated log-normal relaxed clock model (*11*) in a Bayesian Markov chain Monte Carlo framework in BEAST version 1.10 (https://beast.community). HA, hemagglutinin.

The genomic relationship of A/South Australia/85/2018 to IAV-S collected across at least 2 geographically distinct states, Western Australia for the HA gene and Victoria for 5 internal segments, suggests the possibility of IAV-S movement between states in Australia, although IAV-S data for Australia are missing for at least 2–10 years (Figure 1, panel C). This suggestion is, however, contradictory to the data from IAV-S HA collected across 4 states in this study: 10 distinct monophyletic lineages (Figure 2) derived independently from the human pH1N1 lineage, with each group exclusive to 1 state, suggesting that there are spatial restrictions for farms in Australia. 

## Conclusions

A comparison of divergence times between the IAV-S segments from Australia showed that reassortment of endemic viruses with introduced human lineages had been continual (Appendix Figure 3), thereby potentially maintaining sustained transmission on individual swine farms. The risk for emergence of A/South Australia/85/2018-like viruses in humans is potentially high because all 6 internal protein genes are derived from human-adapted pH1N1 virus. The human-origin HA and NA genes of A/South Australia/85/2018 were widely circulating in the human population 20–25 years ago. Hence, children probably have little or no immunity to the HA/NA of this virus, making them more susceptible to infection with this virus subtype, as in the case reported here and in children infected with swine H3N2v virus in the United States (*12*–*14*).

The genomic and antigenic properties and epidemiologic characteristics of zoonotic IAV-S are useful for identifying the potential risk for emergence and spread into the human population. These data also enable better identification of potential nationally relevant mitigation strategies, including measures such as public awareness programs and influenza vaccination of swine herds to eliminate sustained transmission of influenza virus in swine populations (*15*). Our study highlights the risk to the general human population in Australia for infection with IAV-S and the need for more vigilant surveillance of swine and persons who are in close contact with swine to enable early detection and characterization of zoonotic influenza infections.

Appendix Supplementary methods and results from study of locally acquired human infection with swine-origin influenza A(H3N2) variant virus, Australia, 2018.
