# Supplementary material for: Locally Acquired Human Infection with Swine-Origin Influenza A(H3N2) Variant Virus, Australia, 2018
Source: Emerg Infect Dis. 2020 Jan;26(1):143–7. doi: 10.3201/eid2601.191144 (PMC6924914; doi:10.3201/eid2601.191144)
Supplement: Appendix — Supplementary methods and results from study of locally acquired human infection with swine-origin influenza A(H3N2) variant virus, Australia, 2018. [file 19-1144-Techapp-s1.pdf]

Article DOI: https://doi.org/10.3201/eid2601.191144

Locally Acquired Human Infection with Swine-Origin Influenza A(H3N2) Variant Virus, Australia, 2018

Appendix

Methods

Collection of human and swine viruses

The human nasal swab sample was forwarded for further characterization to the WHO Collaborating Centre for Reference and Research on Influenza in Melbourne (WHO CC). Virus isolation was attempted in SIAT-1-MDCK cells (*1*), using standard procedures (*2*). Swine nasal, tracheal, or pooled lung tissue samples were submitted between 2012 and 2018 on an ad hoc basis to the Australian Animal Health Laboratories (AAHL) in Geelong for diagnostic investigations by commercial piggeries in New South Wales (NSW), Queensland (QLD), Victoria (VIC) and Western Australia (WA). Swine IAV (swIAV) isolates were obtained by propagation in MDCK cells (*3*). Haemagglutination titers were obtained on cultures showing cytopathic effects using 0.5% (vol/vol) chicken or 1% (v/v) turkey red blood cells.

Hemagglutinin inhibition Assay

The antigenic relationships of the human and swine IAV were investigated by hemagglutination inhibition (HI) assays using panels of post-infection ferret and swine antisera raised against representative human and swine IAV (see Appendix Table 1 and S2). HI assays were performed with either turkey, guinea pig or chicken red blood cells as previously described using standard techniques (*2*).

Viral genome sequencing and assembly

Viral RNA was extracted using either QIAcube with QIAamp 96 Virus QIAcube HT kit (QIAGEN, https://www.qiagen.com) for human nasal swabs or MagMax Express magnetic particle processor with MagMax-96 viral RNA isolation kit (Thermo Fisher, https://www.thermofisher.com) for swine samples according to the manufacturer’s instructions. Virus subtyping was performed using the CDC Influenza Virus Real-Time RT-

PCR kits provided by the International Reagent Resources (https://www.internationalreagentresource.org) at the WHO CC with SensiFAST Probe Lo-ROX One-Step Kit (Bioline, https://www.bioline.com).

Virus genome segments were amplified by one-step reverse transcription-PCR (RT-PCR) using SuperScript III one-step RT-PCR system with Platinum *Taq* DNA polymerase (Thermo Fisher) and the IAV gene primers with the cycling conditions previously described (*4*). Amplicons were analyzed on TapeStation 4200 (Agilent, https://www.agilent.com) to determine quality and quantity. IAV genomes were sequenced on an IonTorrent PGM (Thermo Fisher) for human IAV, and assembled using FluLINE as previously described (*5*). Genome sequences of swIAV samples were obtained using the Illumina MiSeq (Illumina, https://www.illumina.com) as previously described (*6*).

Phylogenetic analysis

The complete swIAV genome coding sequences determined in this study were compared with sequences obtained from the Influenza Research Database (IRD) and Global Initiative on Sharing All Influenza Data (GISAID) database. BLAST searches of each gene segment of A/SA was used to identify the influenza lineage to which each segment belonged to. Preliminary analysis was conducted using all available H3N2 HA and NA sequences collected during the 1990s and representative sequences from other years, while for the H1N1pdm09 datasets we included 30 sequences from each year from 2009–2018. Following data curation, phylogenetic relationships were estimated for each gene segment independently using the maximum likelihood (ML) method in RAxML v8 (*7*) using the General Time Reversible (GTR) nucleotide substitution model with a gamma (Γ) distribution of among-site rate. Branch support was estimated using a ML bootstrap analysis with replicates ranging from 10 to >1000 replicates for the different datasets analyzed. Time-scaled phylogenies were estimated using the uncorrelated log-normal relaxed clock model (*8*) in a Bayesian Markov Chain Monte Carlo (MCMC) framework in BEAST v1.10 (*9*). Trees were visualized and annotated using FigTree v1.4 (http://tree.bio.ed.ac.uk/software/figtree/).

Accession number(s)

The influenza nucleotide sequences generated from the human case in this study can be obtained using GISAID isolate ID EPI_ISL_341299; and influenza sequences from swine can be obtained from NCBI GenBank using accession numbers MN200947 to MN201152.

References

1. Matrosovich M, Matrosovich T, Carr J, Roberts NA, Klenk HD. Overexpression of the alpha-2,6-sialyltransferase in MDCK cells increases influenza virus sensitivity to neuraminidase inhibitors. J Virol. 2003;77:8418–25. PubMed https://doi.org/10.1128/JVI.77.15.8418-8425.2003

2. World Health Organization. Manual for the laboratory diagnosis and virological surveillance of influenza. Geneva: The Organization; 2011.

3. Swenson SL, Foni E, Saito T, Brown I. Chapter 3.8.7, Influenza A virus of swine. In: Manual of Diagnostic Tests and Vaccines for Terrestrial Animals. Paris: World Organisation for Animal Health; 2019.

4. Zhou B, Donnelly ME, Scholes DT, St George K, Hatta M, Kawaoka Y, et al. Single-reaction genomic amplification accelerates sequencing and vaccine production for classical and swine origin human influenza A viruses. J Virol. 2009;83:10309–13. PubMed https://doi.org/10.1128/JVI.01109-09

5. Zhou B, Deng YM, Barnes JR, Sessions OM, Chou TW, Wilson M, et al. Multiplex reverse transcription-PCR for simultaneous surveillance of influenza A and B viruses. J Clin Microbiol. 2017;55:3492–501. PubMed https://doi.org/10.1128/JCM.00957-17

6. Wong FYK, Donato C, Deng YM, Teng D, Komadina N, Baas C, et al. Divergent human-origin influenza viruses detected in Australian swine populations. J Virol. 2018;92:e00316-18. PubMed https://doi.org/10.1128/JVI.00316-18

7. Stamatakis A. RAxML version 8: a tool for phylogenetic analysis and post-analysis of large phylogenies. Bioinformatics. 2014;30:1312–3. PubMed https://doi.org/10.1093/bioinformatics/btu033

8. Drummond AJ, Ho SY, Phillips MJ, Rambaut A. Relaxed phylogenetics and dating with confidence. PLoS Biol. 2006;4:e88. PubMed https://doi.org/10.1371/journal.pbio.0040088

9. Suchard MA, Lemey P, Baele G, Ayres DL, Drummond AJ, Rambaut A. Bayesian phylogenetic and phylodynamic data integration using BEAST 1.10. Virus Evol. 2018;4:vey016. PubMed https://doi.org/10.1093/ve/vey016

Appendix Table 1. Hemagglutinin inhibition assay using ferret antisera against human and swine H3N2 viruses isolated during 1993–2018.

	**Antisera[1]**											
**Antigen**	A/ Shangdong/ 9/93	A/ Johannesburg/ 33/94	A/ Wuhan/ 359/95	A/ Tasmania/ 1/97	A/ Sydney/ 5/97	A/ Moscow/ 10/99	A/ Minnesota/ 11/2010 (H3N2v)	A/ swine/WA/ 2577766G /2012	A/ Texas/ 50/ 2012	A/Hong Kong/ 4801/ 2014	A/ Singapore/ INFIMH- 16– 0019/2016	A/South Australia/ 85/2018
A/Shangdong/9/93	**1280**	160	40	<20	<20	<20	20	320	<20	<20	<40	<40
A/Johannesburg/33/94	160	**640**	40	<20	20	<20	<20	160	<20	<20	<40	<40
A/Wuhan/359/95	40	20	**640**	80	40	20	<20	80	<20	<20	<40	<40
A/Tasmania/1/97	20	<20	640	**>2560**	20	40	<20	40	<20	<20	<40	<40
A/Sydney/5/97	<20	<20	80	<20	**>2560**	320	20	40	<20	<20	<40	<40
A/Moscow/10/99	<20	<20	<20	<20	>2560	**>2560**	<20	40	<20	<20	<40	40
A/Minnesota/11/2010 (H3N2v)	20	<20	<20	<20	<20	<20	**320**	<20	<20	<20	<40	<40
A/swine/WA/2577766G/2012	40	20	<20	<20	<20	<20	<20	**1280**	<20	<20	<40	80
A/Texas/50/2012	<20	<20	<20	<20	<20	<20	<20	<20	**80**	40	<40	<40
A/Hong Kong/4801/2014	<20	<20	<20	<20	<20	20	<20	<20	80	**320**	40	80
A/Singapore/INFIMH-16–0019/2016	<20	<20	<20	<20	<20	<20	<20	<20	<20	80	**80**	<40
A/South Australia/85/2018	40	<20	<20	<20	<20	<20	<20	40	<20	<20	<40	**1280**

Appendix Table 2. Hemagglutinin inhibition assay using representative swine antisera

	**Antisera[2]**							
**Antigen**	A/sw/Ts/ 4199–1/98	A/sw/Co/ 23619/99	A/sw/Mn/ 01146/06	A/sw/NY/ A01104005/11	A/sw/Iowa/ A01480656/14	A/Sydney/ 5/97	A/Wuhan/ 359/95	A/Moscow/ 10/99
A/swine/Texas/4199–1/1998	**1280**	20	40	<20	20	<20	160	<20
A/swine/Colorado/23619/1999	160	**5120**	1280	40	80	160	160	160
A/swine/Minnesota/01146/2006	80	80	**>5120**	640	80	<20	40	20
A/swine/NewYork/A01104005/2011	40	20	160	**640**	40	<20	<20	<20
A/swine/Iowa/A01480656/2014	160	80	1280	640	**640**	80	320	<20
A/Sydney/5/97	80	640	160	<20	80	**320**	320	40
A/Wuhan/359/95	640	80	320	80	160	80	**1280**	<20
A/Moscow/10/99	80	320	640	<20	<20	160	20	**640**
A/Shangdong/9/93	640	20	160	80	20	<20	320	<20
A/swine/WA/2577766G/2012	320	<20	320	20	20	<20	320	<20
A/swine/WA/2465–2/2016	80	<20	640	20	160	80	80	<20
A/swine/Vic/17–03926/2017	<20	<20	<20	<20	<20	<20	<20	<20
A/South Australia/85/2018	160	20	1280	320	40	40	160	20

Appendix Table 3. Amino acid variation in the H3-HA antigenic epitopes

Virus strain	H3-HA amino acid position (in the antigenic epitopes)						Sequence similarity to A/South Australia /85/2019
	145	155	156	158	189	193	
**Consensus**	**N**	**H**	**X**	**N**	**X**	**N**	
A/South Australia/85/2018		Y	K		S	G	100%
A/swine/WA/2577766G/2012			K	D	S	S	92%
A/swine/WA/2465–2/2016	K		E		R		91%
A/swine/Minnesota/01146/2006			N		R		90%
A/swine/NY/A01104005/2011		Y	N		K		89%

Appendix Table 4. Meta-data of swine IAV sequenced in this study

Virus strain	No. segments sequenced	Collection date	Sample type	Passage details	Genbank accession no.
A/swine/New South Wales/M18–14573–16/2018(H1N1)	8	21/09/2018	Nasal swab	Original	MN200985-MN200992
A/swine/New South Wales/M18–14573–18/2018(H1N1)	1	21/09/2018	Nasal swab	Original	MN200947
A/swine/Queensland/P12–14600–0069–01/2012(H1N1)	8	13/09/2012	Nasal swab	E2	MN200993-MN201000
A/swine/Queensland/P18–01050–0032–01/2018(H1N1)	8	5/03/2018	lung	Original	MN201001-MN201008
A/swine/Queensland/P18–01063–0009–01/2018(H1N1)	8	7/03/2018	Nasal swab	Original	MN201009-MN201016
A/swine/Queensland/P18–04551–0008–01/2018(H1N1)	8	11/09/2018	Nasal swab	MDCK1	MN201017-MN201024
A/swine/Queensland/P18–04551–0011–01/2018(H1N1)	1	11/09/2018	Nasal swab	MDCK1	MN200948
A/swine/Queensland/P18–04551–0012–01/2018(H1N1)	8	11/09/2018	Nasal swab	MDCK1	MN201025-MN201032
A/swine/Queensland/P18–04551–0017–01/2018(H1N1)	1	11/09/2018	Nasal swab	MDCK1	MN200949
A/swine/Victoria/18–04095–0003/2018(H1N1)	8	17/10/2018	Nasal swab	MDCK2	MN201081-MN201088
A/swine/Victoria/18–04095–0006/2018(H1N1)	8	17/10/2018	Nasal swab	MDCK1	MN201089-MN201096
A/swine/Victoria/18–02767–0001/2018(H1N1)	1	10/07/2018	lung	Original	MN200950
A/swine/Victoria/18–02767–0002/2018(H1N1)	8	10/07/2018	lung	Original	MN201073-MN201080
A/swine/Victoria/18–02767–0003/2018(H1N1)	1	10/07/2018	lung	Original	MN200951
A/swine/Victoria/13–1724–1/2013(H1N1)	2	29/08/2013	Nasal swab	Original	MN200952-MN200953
A/swine/Victoria/13–1724–3/2013(H1N1)	2	29/08/2013	Nasal swab	Original	MN200954-MN200955
A/swine/Victoria/13–1724-W1/2013(H1N1)	8	29/08/2013	Nasal swab	Original	MN201033-MN201040
A/swine/Victoria/13–1788–1/2013(H1N1)	8	9/09/2013	Bronchial swab	Original	MN201041-MN201048
A/swine/Victoria/13–1871–2/2013(H1N1)	1	13/09/2013	Nasal swab	Original	MN200956
A/swine/Victoria/13–1871–11/2013(H1N1)	8	13/09/2013	Nasal swab	Original	MN201049-MN201056
A/swine/Victoria/13–1871–15/2013(H1N1)	1	13/09/2013	Nasal swab	Original	MN200957
A/swine/Victoria/13–2493–112/2013(H1N1)	2	16/12/2013	Nasal swab	E1	MN200958-MN200959
A/swine/Victoria/13–2493–124/2013(H1N1)	8	16/12/2013	Nasal swab	Original	MN201057-MN201064
A/swine/Victoria/13–2493-NT78/2013(H1N1)	3	16/12/2013	Nasal swab	E1	MN200960-MN200962
A/swine/Victoria/17–1676–3/2017(H1N1)	8	5/07/2017	lung	E1	MN201065-MN201072
A/swine/Western Australia/AS-13–1125–0016/2013(H1N1)	8	11/04/2013	Pooled tissue	Original	MN201097-MN201104
A/swine/Western Australia/AS-17–3313–0004/2017(H1N1)	1	7/04/2017	Nasal swab	Original	MN200963
A/swine/Western Australia/AS-17–3313–0007/2017(H1N1)	1	7/04/2017	Nasal swab	Original	MN200964
A/swine/Western Australia/AS-17–3313–0009/2017(H1N1)	1	7/04/2017	Nasal swab	Original	MN200965
A/swine/Western Australia/AS-17–3313–0010/2017(H1N1)	1	7/04/2017	Nasal swab	Original	MN200966

Virus strain	No. segments sequenced	Collection date	Sample type	Passage details	Genbank accession no.
A/swine/Western Australia/AS-17–3313–0011/2017(H1N1)	1	7/04/2017	Nasal swab	Original	MN200967
A/swine/Western Australia/AS-17–3313–0013/2017(H1N1)	8	7/04/2017	Nasal swab	Original	MN201105-MN201112
A/swine/Western Australia/AS-17–3313–0020/2017(H1N1)	8	7/04/2017	Nasal swab	MDCK1	MN201113-MN201120
A/swine/Western Australia/AS-17–3313–0001/2017(H1N1)	3	7/04/2017	Nasal swab	Original	MN200968-MN200970
A/swine/Western Australia/AS-17–4797–0001/2017(H1N1)	8	30/05/2017	Nasal swab	MDCK1	MN201121-MN201128
A/swine/Western Australia/AS-17–4797–0005/2017(H1N1)	3	30/05/2017	Nasal swab	Original	MN200971-MN200973
A/swine/Western Australia/AS-17–4797–0006/2017(H1N1)	8	30/05/2017	Nasal swab	MDCK1	MN201129-MN201136
A/swine/Western Australia/AS-17–4797–0010/2017(H1N1)	3	30/05/2017	Nasal swab	Original	MN200974-MN200976
A/swine/Western Australia/AS-17–5267–0001/2017(H1N1)	8	7/07/2017	Lung	MDCK1	MN201137-MN201144
A/swine/Western Australia/AS-18–3511–0001/2018(H1N1)	1	25/09/2018	Nasal swab	Original	MN200977
A/swine/Western Australia/AS-18–3511–0002/2018(H1N1)	1	25/09/2018	Nasal swab	Original	MN200978
A/swine/Western Australia/AS-18–3511–0004/2018(H1N1)	1	25/09/2018	Nasal swab	Original	MN200979
A/swine/Western Australia/AS-18–3511–0005/2018(H1N1)	8	25/09/2018	Nasal swab	Original	MN201145-MN201152
A/swine/Western Australia/AS-18–4235–0002/2018(H1N1)	5	5/11/2018	Lung	Original	MN200980-MN200984

Appendix Table 5. Sequences used in phylogenetic analysis obtained from GISAID*

Isolate ID	Segment	Country	Collection date	Isolate name	Originating Lab	Submitting Lab
EPI398040	HA	United States	2012-Aug-05	A/Ohio/57/2012	Ohio Department of Health Laboratories	Centers for Disease Control and Prevention
EPI393661	HA	United States	2012-Aug-13	A/Ohio/62/2012	Ohio Department of Health Laboratories	Centers for Disease Control and Prevention
EPI575533	HA	Congo	2014-Dec-08	A/Congo/2461/2014	INRB Service de Virologie	Centers for Disease Control and Prevention
EPI651720	HA	United States	2015-Sep-02	A/New Hampshire/38/2015	New Hampshire Public Health Laboratories	Centers for Disease Control and Prevention
EPI346424	HA	United States	2011-Nov-14	A/Iowa/09/2011	Iowa State Hygienic Laboratory	Centers for Disease Control and Prevention
EPI694865	NA	Italy	2014-Jan-08	A/swine/Italy/50064-2/2014	Istituto Zooprofilattico Sperimentale Delle Venezie	Istituto Zooprofilattico Sperimentale Delle Venezie
EPI506844	NA	China	2013-Nov-15	A/Hangzhou/A773/2013	Hangzhou Center for Disease Control and Prevention	Hangzhou Center for Disease Control and Prevention
EPI566710	NA	Myanmar	2013-Jul-22	A/Myanmar/13M089/2013		Niigata University (DPH)
EPI649441	NA	Guatemala	2015-May-03	A/Guatemala/15/2015	Laboratorio Nacional De Salud Guatemala	Centers for Disease Control and Prevention
EPI588529	NA	United States	2015-Mar-08	A/Texas/36/2015	Baylor College of Medicine	Centers for Disease Control and Prevention
EPI397439	NA	United States	2012-Aug-20	A/Wisconsin/30/2012	Wisconsin State Laboratory of Hygiene	Centers for Disease Control and Prevention
EPI579059	NA	Kazakhstan	2015-Jan-08	A/Kazakhstan/39/2015	CSEE	Centers for Disease Control and Prevention

*https://www.gisaid.org.

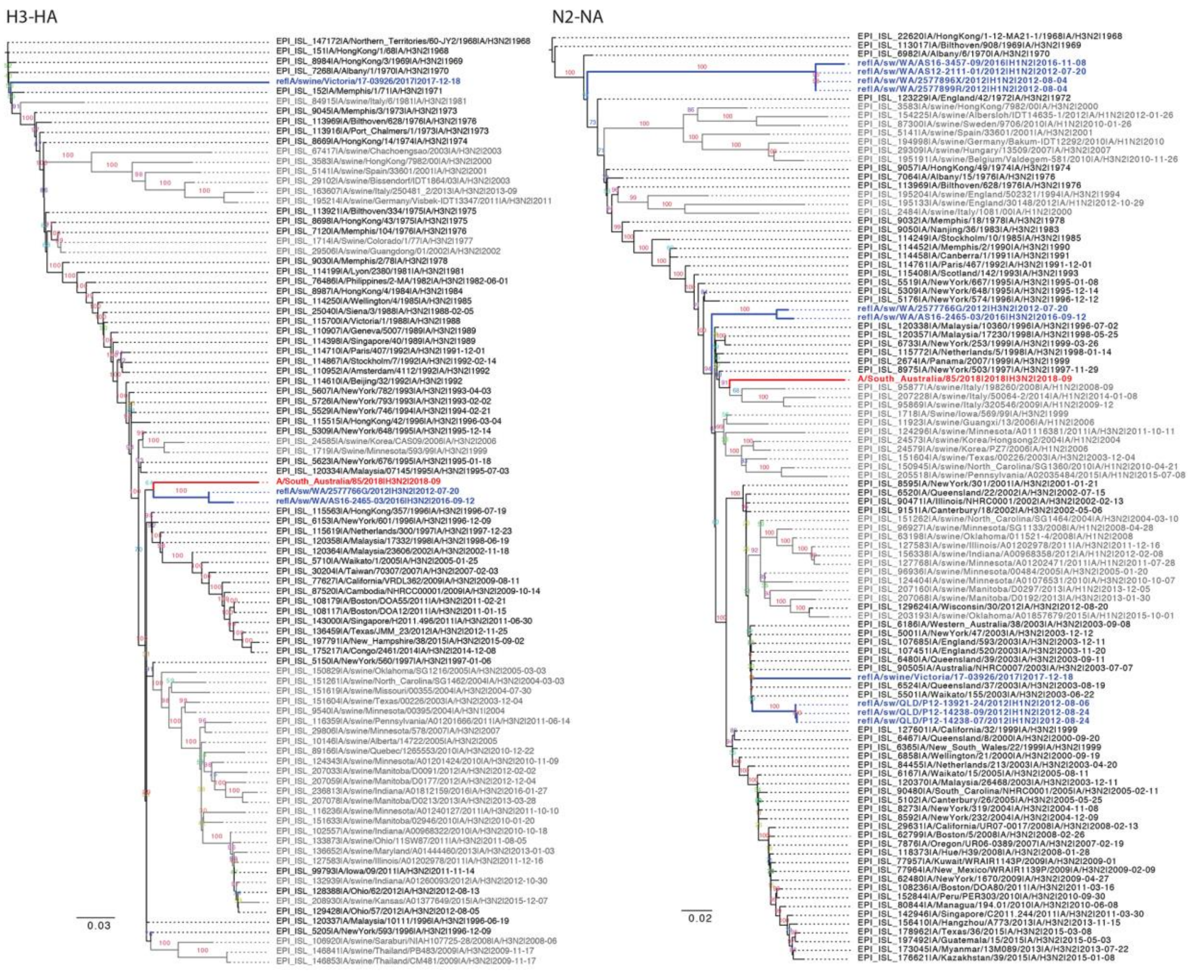

Appendix Figure 1. Evolutionary relationships of the HA and NA genes of A/South Australia/85/2018. Maximum likelihood phylogenies estimated using RAxML v8 [7] showing bootstrap values at branch nodes. Scale-bar represents nucleotide substitutions per site. The human case is colored red, while swIAV collected in Australia are shown in blue.

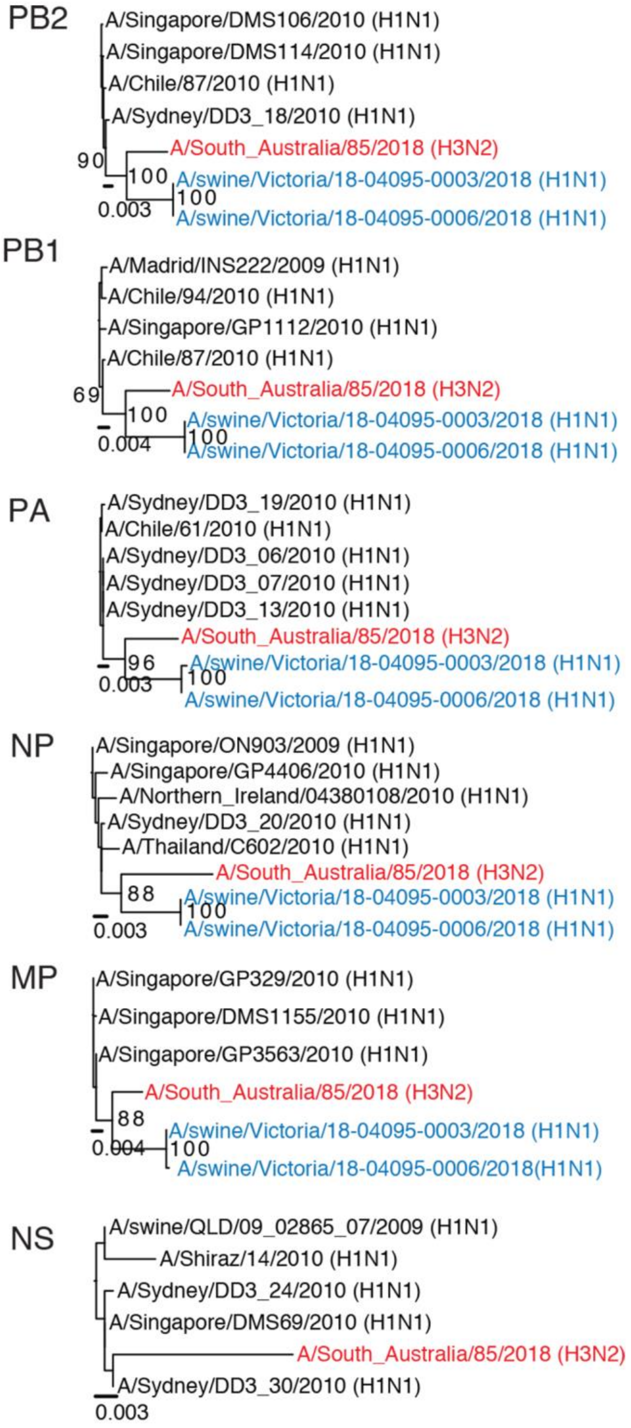

Appendix Figure 2. Origins of the PB1, PA, NP, MP and NS genes of A/South Australia/85/2018. MP, matrix protein; NP, nucleoprotein; NS, nonstructural; PA, polymerase acidic; PB1, polymerase basic 1.

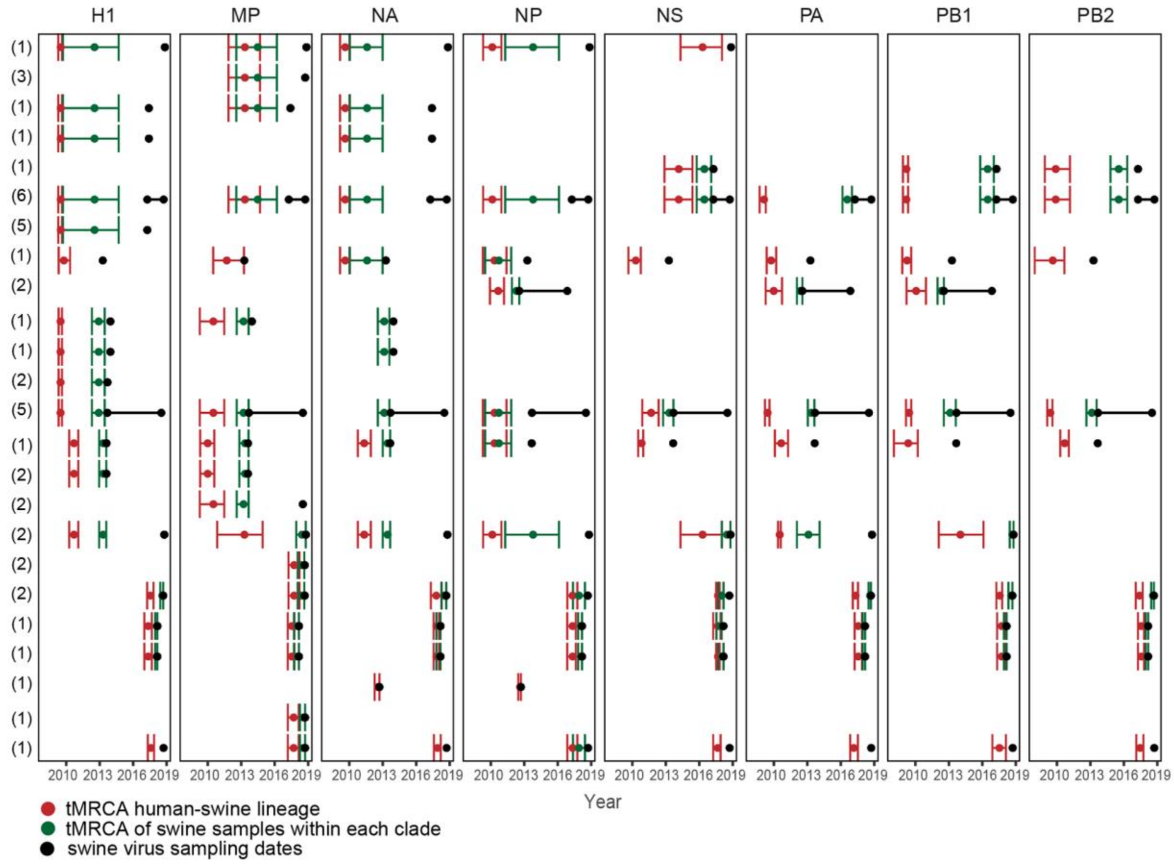

Appendix Figure 3. Origins of Australian swine H1N1pdm09 genes from humans. The mean and 95% confidence intervals of the time of origin of each of the Australian swIAV lineages from human seasonal influenza viruses is shown in red, while the time of divergence of each sub-lineage is shown in green. Numbers denote viruses sharing the same tMRCAs and that formed a similar lineage Divergence times were estimated using the uncorrelated log-normal relaxed clock model in a Bayesian Markov Chain Monte Carlo (MCMC) framework in BEAST v1.10. Trees were visualized and annotated using FigTree v1.4 (See Appendix methods).
